# Dexmedetomidine does not compromise neuronal viability, synaptic connectivity, learning and memory in a rodent model

**DOI:** 10.1038/s41598-021-95635-x

**Published:** 2021-08-09

**Authors:** Nerea Jimenez-Tellez, Fahad Iqbal, Marcus Pehar, Alberto Casas-Ortiz, Tiffany Rice, Naweed I. Syed

**Affiliations:** 1 Department of Biochemistry and Molecular Biology, University of Calgary, Calgary, Canada; 2 Hotchkiss Brain Institute, Calgary, Canada; 3 Alberta Children’s Hospital Research Institute, Calgary, Canada; 4 Department of Cell Biology and Anatomy, University of Calgary, Calgary, Canada; 5 Department of Anesthesiology, Perioperative and Pain Medicine, University of Calgary, Calgary, Canada

**Keywords:** Cellular neuroscience, Learning and memory, Cell death

## Abstract

Recent animal studies have drawn concerns regarding most commonly used anesthetics and their long-term cytotoxic effects, specifically on the nervous tissue. It is therefore imperative that the search continues for agents that are non-toxic at both the cellular and behavioural level. One such agent appears to be dexmedetomidine (DEX) which has not only been found to be less neurotoxic but has also been shown to protect neurons from cytotoxicity induced by other anesthetic agents. However, DEX’s effects on the growth and synaptic connectivity at the individual neuronal level, and the underlying mechanisms have not yet been fully resolved. Here, we tested DEX for its impact on neuronal growth, synapse formation (in vitro) and learning and memory in a rodent model. Rat cortical neurons were exposed to a range of clinically relevant DEX concentrations (0.05–10 µM) and cellular viability, neurite outgrowth, synaptic assembly and mitochondrial morphology were assessed. We discovered that DEX did not affect neuronal viability when used below 10 µM, whereas significant cell death was noted at higher concentrations. Interestingly, in the presence of DEX, neurons exhibited more neurite branching, albeit with no differences in corresponding synaptic puncta formation. When rat pups were injected subcutaneously with DEX 25 µg/kg on postnatal day 7 and again on postnatal day 8, we discovered that this agent did not affect hippocampal-dependent memory in freely behaving animals. Our data demonstrates, for the first time, the non-neurotoxic nature of DEX both in vitro and in vivo in an animal model providing support for its utility as a safer anesthetic agent. Moreover, this study provides the first direct evidence that although DEX is growth permissive, causes mitochondrial fusion and reduces oxygen reactive species production, it does not affect the total number of synaptic connections between the cortical neurons in vitro.

## Introduction

Anesthetic agents are commonly used in patients undergoing various surgical procedures^1–3^ and their safety and efficacy has improved significantly over time^4^. Notwithstanding the safety of the newer compounds, evidence is emerging to suggest that numerous clinically used anesthetics may cause cytotoxicity^5^—especially when tested on animal models. The evidence of harm in the context of humans however remains polemical^6^. This information is particularly important in the context of both developing and aging brains when neurons rely upon their activity and connectivity for communications and the formation of new synapses underlying learning and memory. Blocking activity or altering neuronal activity patterns, leads either to a reduction in synaptic strength or to the elimination of neuronal activity altogether^7^. Since most anesthetics exert their actions by suppressing neuronal activity and shutting off synaptic communications, it is believed that their neurotoxic effects likely involve both structures and functions that are deemed essential for neuronal excitability. Specifically, any compound that inhibits electrical activity during the critical time window encompassing synaptogenesis is likely to alter the network’s assembly. For instance, blocking electrical activity during development severely compromises not only neuronal projections but also synaptic connectivity^8^. It therefore stands to reason that blocking neuronal activity with anesthetics, specifically when synaptic connections are being formed *en masse* may affect brain development and connectivity. However, it remains to be determined whether the anesthetic-induced perturbation of synaptic connectivity has short- or long-term effects or if synaptic plasticity-dependent mechanisms may compensate for the loss of function^9,10^. Several studies have shown that the blockage of ion channels^11^, electrical activity^10^ and transmitter-receptor interactions^12^ have detrimental effects both on neural projections and synaptic connectivity^5^. It is then reasonable to presume that anesthetics action upon these potential target sites may also affect neuronal input and output functions and the underlying synaptic structures and function.

While a complete understanding of the neurotoxic mechanisms underlying these potential deficits in humans is yet to be deduced, recent research has shown that most commonly used anesthetics, such as sevoflurane, propofol and ketamine, are neurotoxic in various animal models^5^. Consequently, these agents are now being supplemented with a number of other compounds in anticipation that they might stem the anesthetic-induced neurotoxicity^5^, though the evidence and the underlying mechanisms remain to be fully defined.

One promising agent in this regard is the α2-adrenergic receptor agonist dexmedetomidine (DEX)^13^. This is of relevance because these receptors are broadly distributed across the nervous system and are important for the regulation of adrenaline and noradrenaline^9^ mediated synaptic transmission. Unlike contemporary anesthetics that act primarily as *N*-methyl-d-aspartate (NMDA) receptor antagonists or γ-aminobutyric acid (GABA) receptor agonists^5,14^, DEX inhibits norepinephrine release downstream of α_2_-adrenergic receptor activation^15^. DEX specifically targets norepinephrine pathways in the locus coeruleus mirroring non-rapid eye movement (NREM) sleep^16^. DEX-induced sedation is thus markedly different at the cellular level from other anesthetics as it mimics the endogenous sleep pathway. Although DEX is now being used clinically in the pediatric population, its cytotoxic potential has not been extensively tested in animal models in a manner analogous to that of other anesthetics. Current literature has focused on the effects of DEX on cell death in situ*,* mainly testing its impact on cellularly viability in the hippocampal region using apoptotic markers such as cleaved caspase 3 expression levels ^17^, BAX/Bcl2 ratio^18^, TUNEL staining^19^ or PI3K/AKT/GSK3β^18,20^. However, in instances where it has been tested for its anti-apoptosis role in situ, the data is often elusive or controversial^21,22^. Furthermore, in most of these studies, the role of DEX on its own has not been investigated. Rather this agent is most often exploited as a neuroprotective agent when used as an adjuvant with other conventional anesthetics that are found to be neurotoxic^20,23^. These studies do not provide unequivocal evidence regarding DEX-mediated protective effects as the brain tissue utilized was often collected following euthanasia induced by different anesthetic agents thus generating confounding results^24,25^. Moreover, the studies conducted so far have focused primarily on the effects of DEX in vivo involving the complexity of the whole organism^26–28^ and not in the context of synaptic viability/plasticity at the level of individual neurons. Additionally, the DEX-induced effects have either been examined over a shorter time period^29^ or at one time point^30^, and they have also failed to identify the potential target sites for DEX-mediated effects^31^.

Given the questions that remain unanswered about the specific effects of DEX and the numerous potential clinical applications of this agent, it is imperative to clearly elucidate any neurotoxic effects this agent may have. In an attempt to fully examine the effects of DEX on cellular homeostasis, viability, growth and synaptic assembly, we explored a range of different concentrations of this agent in vitro using rat cortical neurons. To determine whether DEX exposure compromises learning and memory in vivo, postnatal day 7 pups were injected with DEX and later subjected to various learning and memory tasks.

## Materials and methods

### Experimental animals

Postnatal day 0 wild-type Sprague Dawley (strain code 400) rats from Charles River Laboratories were used in this study. Rats were kept in a conventional room set at 25 °C on a 12 h-light/dark cycle from 7 am to 7 pm and rat got fed ad libitum*.* Sedation of these pups was accomplished via ice-induce hypothermia, where the postnatal day 0 pups were wrapped in tissue paper and placed in a container filled with ice for 7 min^35^. Following the loss of movement and preceding the regaining of consciousness, decapitation was performed to achieve euthanasia in the pups. Cortical tissue was collected right after decapitation.

### Primary rat neuronal cell culture and dexmedetomidine treatment

Sprague–Dawley rat frontal cortices were isolated and cultured as previously described^34^. Some cultures were treated with various concentrations of dexmedetomidine (10 mg; MilliporeSigma, St. Louis, MO, USA) (0.05 μM, 0.1 μM, 1 μM, 2.5 μM, 5 μM, or 10 μM) dissolved in culture media, whereas controls only had culture media. These concentrations were chosen for a dose–response assay based on previous literature and clinical equivalents^22,36^.

### Cell viability assay

The effects of DEX on neuronal viability were tested 3 days post-culture via the LIVE⁄DEAD Viability/Cytotoxicity Kit (Molecular Probes) as previously described^34^. In order to assess the effect at a later time point, we also analyzed the viability of the remaining alive cells at day seven post-exposure. Cells were imaged on a Zeiss Axio Observer Z1 microscope (Zeiss Corp.) using a 10X/0.3 Ph1 DICII objective.

### Live-cell fluorescent imaging and confocal microscopy

To evaluate the impact of DEX on the morphological integrity of the mitochondria, cells were grown for 4, 7 and 10 days post-culture and then their media replaced with MitoTracker Green (ThermoFisher Scientific, cat. M7514) (25 nM)—containing media (37 °C and incubated with the cells for 12 min at 37 °C and 5% CO_2_). The cells were then washed three times with warm Dulbecco's phosphate-buffered saline (DPBS), and fresh media was added prior to imaging. Cells were visualized under the microscope and imaged. Fluorescence images were taken on an Olympus SD-OSR spinning-disk confocal microscope (100×/1.49 oil)) with a mounted incubator system (Olympus Corp.) and all imaging parameters were kept identical for each dish. Mitochondrial morphology was quantified as previously described^34^.

### Reactive oxygen species (ROS) production using flow-cytometry

ROS production was quantified over time (Days 1, 2, 3, 4, 7 and 10 following experimental treatment). A procedure derived from de Brito Monteiro et al.^37^ was adopted. For our study, the cells of interest were unfixed rat cortical neurons that were cultured in 6-well plates and harvested using 0.2 mL of Trypsin/EDTA 0.25% (ThermoFisher Scientific, cat. 252000556) per well at 37 °C for 7 min and neutralized with 2 mL of 10% FBS (Thermo Fisher Scientific, Waltham, MA, USA) in Hank’s Balance Salt solution HBSS (ThermoFisher Scientific, cat. 88,284). The markers used in our experiments were 2 µM Calcein AM from Live/Dead Viability/Cytotoxicity kit (ThermoFisher Scientific, cat. L3224) and 5 µM MitoSox Red (ThermoFisher Scientific, cat. M36008). The apparatus used for cell sorting was BD LSR II (BD Biosciences, USA) and the software used for data analysis was FlowJo (BD Biosciences).

### Localization of neuronal/synaptic morphology via immunocytochemistry

Immunofluorescent staining was performed to assess the impact of dexmedetomidine on neuronal cytoskeletal growth in neurons grown for 7 days post-culture. Fixation and staining were done as previously described for neurofilament antibody^34^. Cells were imaged on a Zeiss Axio Observer Z1 microscope (Zeiss Corp.) using a 20×/0.8 DICII objective. Fifteen to twenty neurons per biological replicate (n = 12–13) were quantified.

A procedure similar to the one previously outlined was followed to evaluate the impact of DEX on synaptic network assembly in neurons grown for 7 days post-culture using synaptophysin and PSD-95 antibodies as previously described^34^. Cells were imaged on a Zeiss Axio Observer Z1 microscope (Zeiss Corp.) using a 63×/1.4 oil DICII objective 15–20 neurons per biological replicate (n = 8–10) were quantified.

### Dexmedetomidine treatment

Sprague–Dawley pregnant rats were purchased from a commercial breeder (Charles River Laboratories, Senneville, QC, Canada). Gestational progress was monitored from E16.5 until the date of birth (post-natal day zero—P0).

P7 rat pups of both sexes were injected with 25 µg/kg of dexmedetomidine subcutaneously either once (1×) or twice (2×), (24 h after the first injection), using a 0.1 ml volume. Control animals were injected with the same volume of saline (sodium chloride 0.9% B Braun, Mississauga, ON, Canada). The core body temperature of all animals was maintained with the use of a heated blanket and gloves filled with warm water replaced every 20 min. The respiratory status of the animal was monitored by counting breaths per minute monitored every 10 min following the first injection for the following 30 min, to ensure the pup’s health. Furthermore, oxygen saturation (SpO_2_) was monitored every 30 min after the first injection for the period of an hour to further ensure the pup’s health and reception to the compound (Nonin Medical, Minneapolis, MN, USA).

Each pup’s ear was notched at P14 and pups were weaned in cages of 2–3 animals, separated by sexes. Animals were kept undisturbed until P60 and then subjected to various behavioural tests.

### Morris water maze

A 1.8 m diameter pool of water with a platform hidden 1 cm below the water surface (25 °C) was placed in a room that had remote visual cues on four different walls. A P60 rat was placed in the water first at the quadrant closest to the hidden platform (Q1) facing the pool walls and left to swim in the water until it reached the platform or not within an allocated 60 s period. If the animal had found the platform, it was left there for 15 s and then placed back into an empty cage with bedding for 45 s before placing it in the next 3 quadrants (Q2, Q3 and Q4). If the animal did not reach the platform before 60 s, the software tracked the latency as 60 s. This process was repeated for 5 consecutive days and latency (time taken) to reach the platform and distance swum were measured by the automated software, ANY-Maze (Stoelting Co, Wood Dale, IL, USA). Latency and distance were used as the learning and memory parameters characterized over the period of days 1 and 5.

### Novel object recognition

Object selection was carried out according to established parameters that allow rats to discriminate between objects of similar complexity to avoid potential preference that might otherwise compromise the results^38^.

For 2 days, the P67 animals went through the first phase, habituation, which consisted of the placement of an animal in a square empty box of 60 cm per side with bedding on the floor and left to interact with the new environment for 15 min per day. On the third day, two identical objects were placed diagonally in the box and the animals were left to interact with these objects for 5 min during the familiarization phase. The animals were then taken from the box and placed in an empty cage for a 5 min retention interval. In the final step, testing was assessed for 3 min by replacing one of the familiar objects with a novel one and the ratio of the time spent interacting with the new object was monitored and measured to inquire on recognition learning and memory. To avoid bias, the object replaced to be the novel object changed for each animal (e.g. Object 2 for even-numbered animals and Object 1 for odd-numbered animals). On the final day (day 4), the last three steps were repeated however, instead of replacing one object with a novel object, one of the familiar objects was placed in a different location in the box. Similarly, the object moved in the box was changed for each animal to avoid bias.

### Statistical analysis

All samples were assigned randomly, and the experiments were performed in a single-blinded fashion. Specifically, the observer was unaware of the experimental conditions. Statistical significance tests were conducted with GraphPad Prism 8. One-way ANOVA was used to compare between multiple groups, followed by Dunnett’s or Tukey’s multiple comparisons tests for post hoc comparisons. Differences between the means of two conditions were tested using the two-sided Student’s t-test with Welch’s correction. Differences between data were considered significant if appropriate post-hoc statistical tests resulted in p ≤ 0.05.

### Ethical statements

All animal procedures were carried out in compliance with the standards established by the University of Calgary Animal Care and Use Policy under the Canadian Council on Animal Care. All experimental procedures were approved by the University of Calgary Animal Care Committee (protocol AC21-0032). The most suitable species were chosen for this study, and data were obtained in accordance with the best existing practices that ensure the animals’ welfare^32^. The protocols used in this study are essentially identical to those employed in previous studies^33,34^ and were adapted suitably. All in vivo experiments was carried out in accordance to ARRIVE guidelines.

## Results

### DEX exposure did not affect cell viability

**Figure 1:**
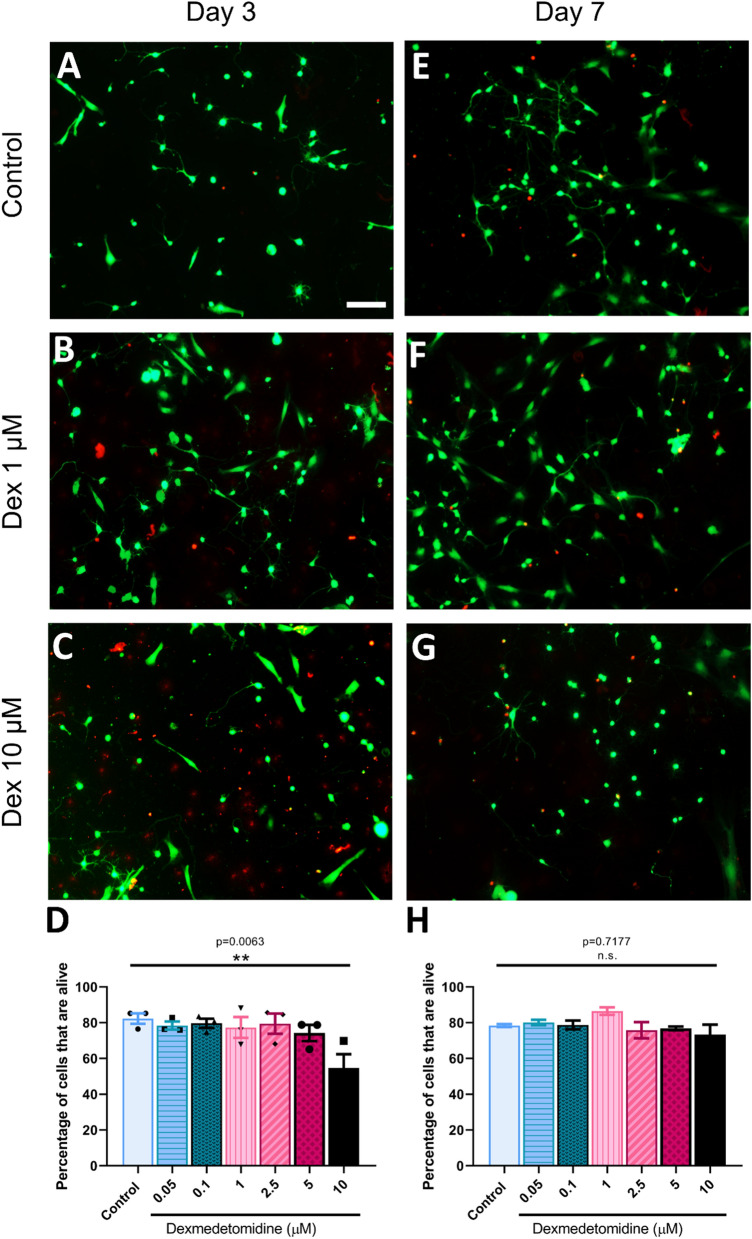
Dexmedetomidine did not compromise cell viability. Representative live-fluorescent images of different conditions with a LIVE/DEAD viability/cytotoxicity assay where live cells are labeled with calcein-AM (green) and dead cells are labeled with ethidium homodimer-1 (red). (**A**) Control cells, (**B**) cells exposed to dexmedetomidine 1 μM and (**C**) dexmedetomidine 10 μM at day 3 after DEX exposure and (**E**) control cells, (**F**) cells exposed to dexmedetomidine 1 μM and (**G**) dexmedetomidine 10 μM at day 7 after DEX exposure. Scale bar indicates 100 μm. (**D**) Quantification of the percentage of cells that are alive at day 3: control = 82.3 ± 2.929, Dex 0.05 = 78.37 ± 2.254, Dex 0.1 = 79.64 ± 2.606, Dex 1 = 77.3 ± 5.868, Dex 2.5 = 79.45 ± 5.656, Dex 5 = 74.23 ± 4.539 and Dex 10 = 54.74 ± 7.638. Values are mean ± SEM F (6, 14) = 3.673, **p = 0.0063 by one-way ANOVA with Dunnet’s post hoc analysis for multiple comparisons. (**H**) Quantification of the percentage of cells that are alive at day 7: control = 78.34 ± 0.824, Dex 0.05 = 80.11 ± 1.532, Dex 0.1 = 78.74 ± 2.485, Dex 1 = 86.46 ± 2.075, Dex 2.5 = 75.79 ± 4.487, Dex 5 = 76.76 ± 1.06 and Dex 10 = 73.34 ± 5.497. Values are mean ± SEM F (6, 14) = 1.85, p = 0.7177 by one-way ANOVA with Dunnet’s post hoc analysis for multiple comparisons. Concentrations of dexmedetomidine expressed in μM. Bars indicate ± SEM n = 3 dishes per condition, 15–20 images per plate.

In this study, we first sought to confirm whether various clinically used concentrations of DEX impacted cellular viability, and if these effects were dose-dependent. To avoid confounders of the whole animal or intact brain slices, we used a well-established in vitro cell culture model^33^. 1 to 2.5 μM has been recommended as a clinically relevant DEX concentration for in vitro experiments, and 5 μM deemed to be a higher dose in the clinical domain^39^. In the present study, we tested for the first time, all of the above concentrations to obtain a more comprehensive dose–response curve. Multiple areas (0.58 mm^2^) of three replicates of each condition were imaged and quantified using one-way ANOVA testing, with identical thresholds at days 3 (Fig. 1A–C) and 7 (Fig. 1E–G) after DEX exposure. We found that DEX did not impact cellular viability at concentrations below 10 μM (Fig. 1). However, at 10 μM, DEX significantly reduced cellular viability, resulting in greater cell death (mean = 54.74%, SEM = 7.638 alive p = 0.0063, n = 3) compared to the control (mean = 82.3, SEM = 2.929, n = 3) at day 3 after exposure (Fig. 1D). At day 7, DEX did not impact cellular viability any further when tested in all of the above concentrations (Fig. 1H). Collectively, these results suggest that DEX is non-cytotoxic—that is, it did not induce cell death in newly cultured, neonatal rat neurons at clinically relevant concentrations. Our dose response curve suggested that DEX at 1 μM was a safe and perhaps clinically relevant concentration, so all subsequent experiments from hereon were conducted with this concentration.

### DEX administration promoted neuronal outgrowth

**Figure 2:**
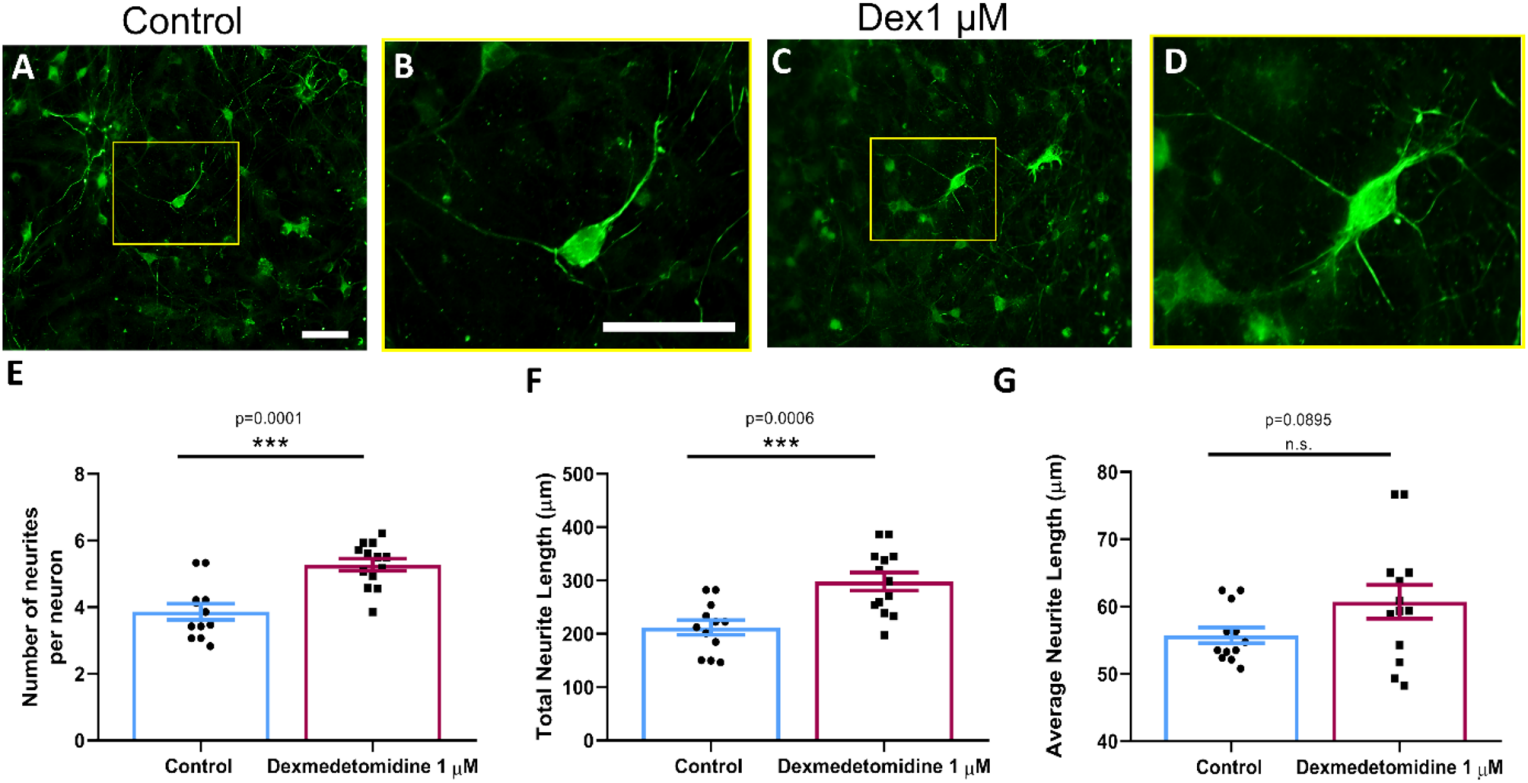
Exposure to 1 μM dexmedetomidine modified neuronal morphology. Representative fluorescent images of neurons stained with neurofilament. (**A**) Control cells and (**B**) higher magnification of the region of interest and (**C**) dexmedetomidine 1 μM treated cells and (**D**) higher magnification of the region of interest. Quantification of (**E**) number of neurites per neuron: control = 3.86 ± 0.239, dexmedetomidine 1 μM = 5.27 ± 0.185. Values are mean ± SEM t = 4.672, df = 21.18, ***p = 0.0001 by two-tailed t test, Welch-corrected (**F**) total neurite length: control = 211.9 ± 13.81, dexmedetomidine 1 μM = 298.0 ± 16.82. Values are mean ± SEM t = 3.956, df = 22.48, ***p = 0.0006 by two-tailed t test, Welch-corrected and (**G**) average neurite length: control = 55.74 ± 1.185, dexmedetomidine 1 μM = 60.71 ± 2.492. Values are mean ± SEM t = 1.8, df = 17.09, P = 0.0895 by two-tailed t test. Bars indicate ± SEM. Scale bars indicate 50 μm n = 12 (control) and n = 13 (dexmedetomidine 1 μM) dishes per condition, 15–20 images per plate. (**A**,**C**,**B**,**D**) show the same scale, respectively.

To assess whether DEX suppressed (like most other anesthetics) or impacted the total extent of neurite outgrowth, we tested its effect on developing rat cortical neurons in vitro. Specifically, we asked whether the growth of newly cultured neurons would be impacted by DEX. The cultured neurons were fixed and stained with polyclonal antibodies against the 160 kDa fragment of neurofilament (Fig. 2) and the extent of total neurite lengths was quantified in multiple areas of 0.15 mm^2^, using two-tailed t tests. We found that cells exposed to 1 μM DEX (Fig. 2C,D) had more neurite processes emanating from the cell body (mean = 5.274, SEM = 0.185, p = 0.0001, n = 13) compared to their control counterparts (Fig. 2A,B) (mean = 3.864, SEM = 0.239, n = 12) (Fig. 2E). We also observed that the total neurite length per cell was greater for DEX treated cultures (mean = 298.0, SEM = 16.82, p = 0.0006, n = 13) as compared to their controls (mean = 211.9, SEM = 13.81, n = 12) (Fig. 2F). We did not however, observe significant differences in the average neurite length (control: mean = 55.74, SEM = 1.185, n = 12 and DEX: mean = 60.71, SEM = 2.492, p = 0.0895, n = 13) (Fig. 2G). Altogether, these results demonstrate that DEX did not affect neuronal ability to initiate growth, but rather cells exposed to this agent exhibited more branching per neuronal process as compared with their control counterparts.

### DEX treatment did not impact synaptic formation

**Figure 3:**
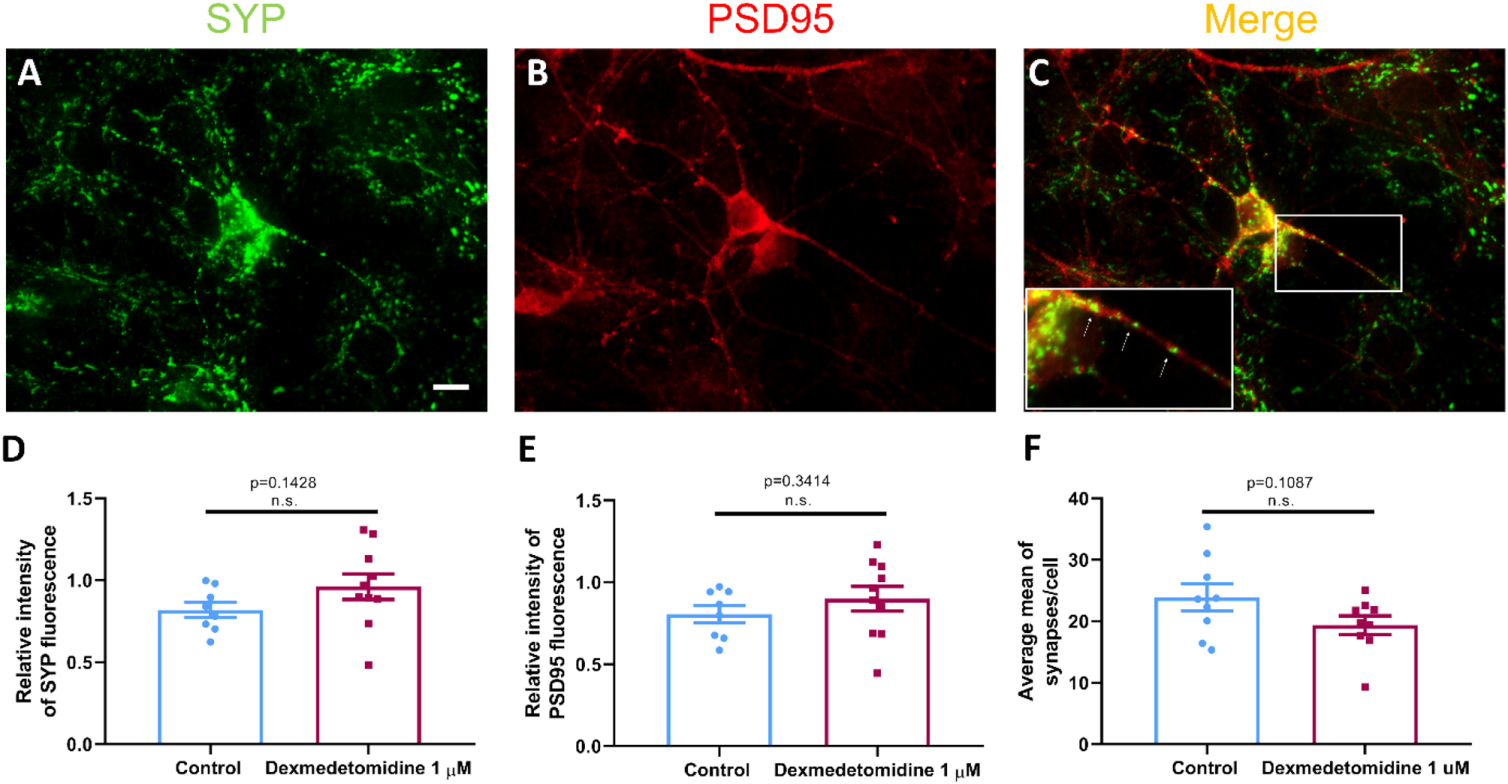
Exposure to 1 μM dexmedetomidine did not affect synapse formation. Representative fluorescent images of neurons stained with SYP (green) and PSD95 (red). (**A**) SYP and (**B**) PSD95 images of control cells. (**C**) SYP/PSD95 merge with close-up look at a synaptic puncta. Quantification of (**D**) relative intensity of SYP fluorescence: control = 0.82 ± 0.048 (n = 8), dexmedetomidine 1 μM = 0.961 ± 0.078 (n = 10). Values are mean ± SEM t = 1.551, df = 14.32, p = 0.143 by two-tailed t test, Welch-corrected and (**E**) relative intensity of PSD95 fluorescence: control = 0.805 ± 0.053 (n = 8), dexmedetomidine 1 μM = 0.89 ± 0.076 (n = 10). Values are mean ± SEM t = 1.025, df = 15.24, p = 0.341 by two-tailed t test, Welch-corrected and (**F**) average number of synapses per cell: control = 23.9 ± 2.17 (n = 9), dexmedetomidine 1 μM = 19.37 ± 1.517 (n = 9). Values are mean ± SEM t = 1.711, df = 14.31, p = 0.1087 by two-tailed t test, Welch-corrected. Bars indicate ± SEM. Scale bars indicate 10 μm.

We next asked whether neurons exhibiting growth also established synaptic connections as defined by the juxtaposition of their presynaptic and postsynaptic proteins that are the hallmark of synaptic specialization. The cells cultured either in the absence or presence of DEX were fixed on day 7 and stained for two relevant synaptic proteins—synaptophysin (SYP) (Fig. 3A), a presynaptic vesicle protein, and postsynaptic density protein (PSD-95) (Fig. 3B). First, we assessed the relative levels of fluorescent intensity for both proteins in control and DEX-treated cells and analyzed the data using two-tailed t tests, Welch-corrected. We did not find significant changes in the expression levels for either SYP (control: mean = 0.8199, SEM = 0.0478, n = 8 and DEX: mean = 0.961, SEM = 0.078, p = 0.143, n = 10) (Fig. 3D) or PSD-95 (control: mean = 0.805, SEM = 0.053, n = 8 and DEX: mean = 0.8998, SEM = 0.076, p = 0.321, n = 10). (Fig. 3E). Second, in order to determine synaptic specialization and formation of neuronal connections, we quantified the boutons where both pre- and post-synaptic proteins were expected to be in juxtaposition (Fig. 3C). Specifically, we measured the total number of synaptic puncta across the entire neurite length of a neuron, both in control and DEX cells. The control and DEX-treated neurons exhibited uniform expression of SYP (green) puncta in juxtaposition with PSD-95 (red) (control: mean = 23.9, SEM = 2.17, n = 9 and DEX: mean = 19.37, SEM = 1.517, p = 0.109, n = 9) (Fig. 3F). Taken together, these data suggest that despite the increase in the length of neurite outgrowth/branching, there was no significant change in the total number of synaptic puncta per cell. These data demonstrate that, although DEX may have enhanced the length and the extent of growth, the total number of synaptic connections made between any two given cells did not change significantly.

### DEX exposure resulted in a hyperfused mitochondrial network

**Figure 4:**
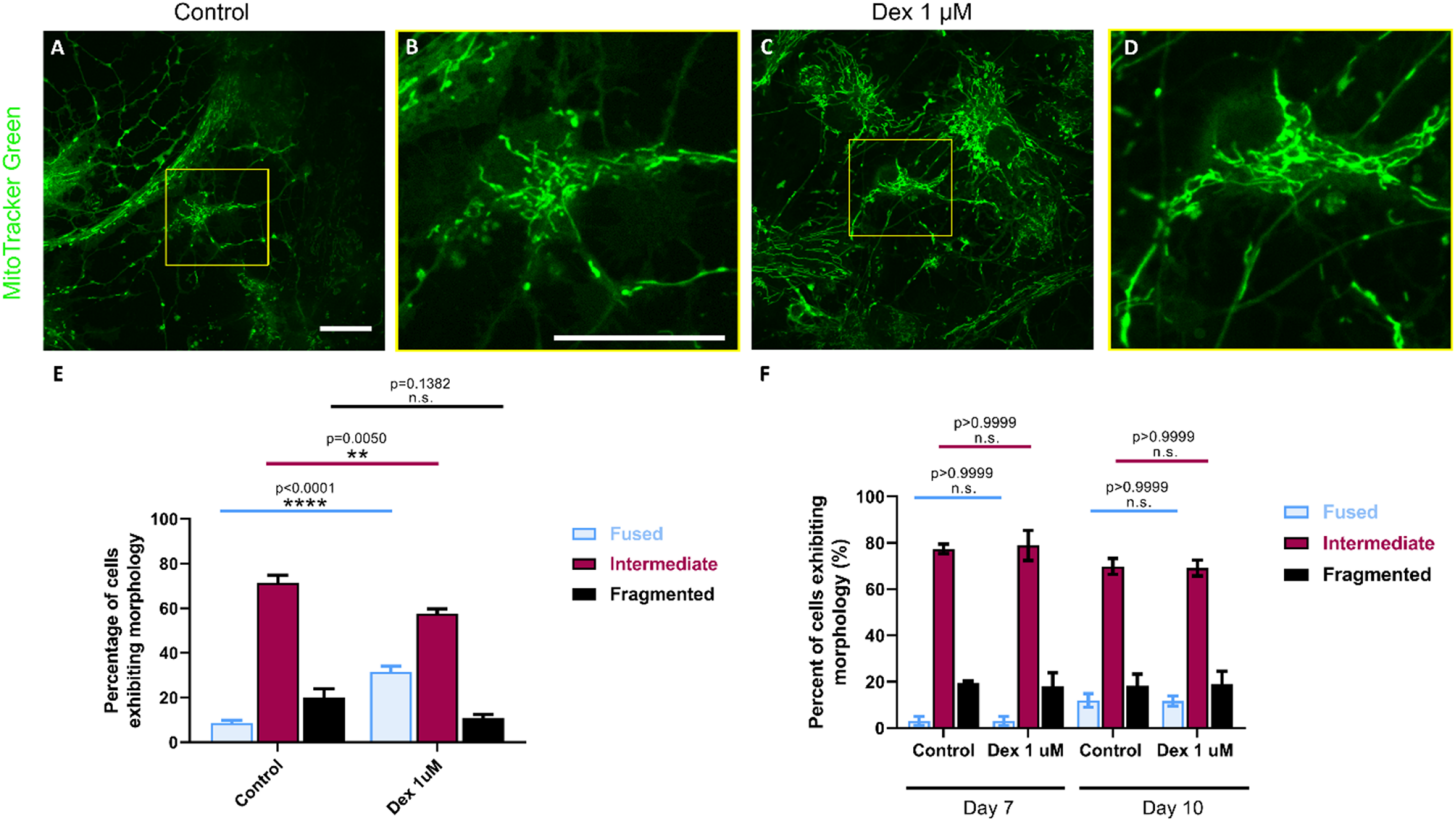
Exposure to dexmedetomidine 1 μM resulted in a hyperfused mitochondrial network. Representative live-fluorescent images of neurons stained with MitoTracker green (**A**) control and (**B**) dexmedetomidine 1 μM. Magnification of the region of interest in (**C**) control and (**D**) dexmedetomidine 1 μM cells. (**E**) Day 4 quantification of predominant mitochondrial morphology in each treatment based on a previously described morphology scale: (1) fused: control = 8.519 ± 1.357, dexmedetomidine 1 μM = 31.65 ± 2.460, (2) intermediate: control = 71.45 ± 3.439, dexmedetomidine 1 μM = 57.53 ± 2.254 and (3) fragmented: control = 20.03 ± 3.941, dexmedetomidine 1 μM = 10.82 ± 1.52. Values are mean ± SEM F (2, 48) = 30.5, ****p < 0.0001, **p = 0.005 by one-way ANOVA with Tukey’s post hoc analysis for multiple comparisons. Bars indicate ± SEM. Scale bars indicate 20 μm n = 8 (control) and n = 10 (dexmedetomidine 1 μM) dishes per condition, 15–20 images per plate. (**F**) Days 7 and 10 quantification of predominant mitochondrial morphology in each treatment: (1) fused: control D7 = 3.077 ± 1.942, control D10 = 11.93 ± 2.94, dexmedetomidine 1 μM D7 = 3.04 ± 2.032, dexmedetomidine 1 μM D10 = 11.72 ± 2.15, (2) intermediate: control D7 = 77.41 ± 2.039, control D10 = 69.8 ± 3.455, dexmedetomidine 1 μM D7 = 78.85 ± 6.503, dexmedetomidine 1 μM D10 = 69.14 ± 3.401 and (3) fragmented: control D7 = 19.51 ± 0.8, control D10 18.27 ± 4.989, dexmedetomidine 1 μM D7 = 18.11 ± 5.725, dexmedetomidine 1 μM D10 = 19.14 ± 5.416. Values are mean ± SEM F (6, 39) = 1.314, P = 0.274 by two-way ANOVA with Tukey’s post hoc analysis for multiple comparisons. Bars indicate ± SEM n = 3 (control and dexmedetomidine 1 μM D7), n = 4 (control D10) and n = 7 (dexmedetomidine 1 μM D10) dishes per condition, 15–20 images per plate. (**A**,**C**,**B**,**D**) show the same scale, respectively.

Neuronal growth and synaptic assembly are high energy demanding processes that require intense mitochondrial efficacy. Previous studies have demonstrated that the mitochondria are a potential site for anesthetic-induced toxicity^5^, accordingly, we next asked whether DEX-mediated effects on cellular viability and growth may also have involved this organelle. In order to test the effects of DEX on mitochondrial morphology, we conducted live-cell imaging using MitoTracker Green to specifically monitor mitochondrial dynamics and subsequently analyzed this data using two-way ANOVA. Each individual neuron was assigned a distinct mitochondrial “identifier” based on their total numbers and the morphology that was specific to any given cell. This criteria for mitochondrial morphology characterization was based on previously established studies where they are classified either as fragmented when less than 0.75 μm in length, intermediate when 0.75–3 μm in length, and fused when greater than 3 μm in length^34^. We observed that the cells exposed to DEX had hyperfused mitochondrial networks at day 4 post-exposure (Fig. 4C,D) (mean = 31.65%, SEM = 2.46 fused mitochondria, p < 0.0001, n = 10), compared to the control cells (Fig. 4A,B) (mean = 8.519%, SEM = 1.357 fused mitochondria, n = 8), and this was in complete contrast to what has previously been reported for other anesthetics^33,40,41^ (Fig. 4E). In those instances where we did observe a fraction of cells showing fragmented morphology, these data were not statistically significant (control: mean = 20.03, SEM = 3.941, n = 8 and DEX: mean = 10.82, SEM = 1.520, p = 0.138, n = 10). To assess if this hyperfusion occurred in an acute manner, or was maintained over time, we analyzed mitochondrial morphology at days 7 and 10 post-exposure. We found that the hyperfused mitochondrial morphology was not maintained over time as by day 7 and day 10, neither was the fused nor the intermediate fraction of DEX-exposed cells were significantly different from the control (Fig. 4F). Taken together, these results demonstrate that DEX likely acts as a “switch” enabling the intermediate mitochondrial fraction to exhibit a more fused form in a time-dependent manner, which may likely enhance the energy production capacity of the cell mediating its positive effects.

### DEX treatment resulted in a decrease in ROS production at seven days post-exposure

**Figure 5:**
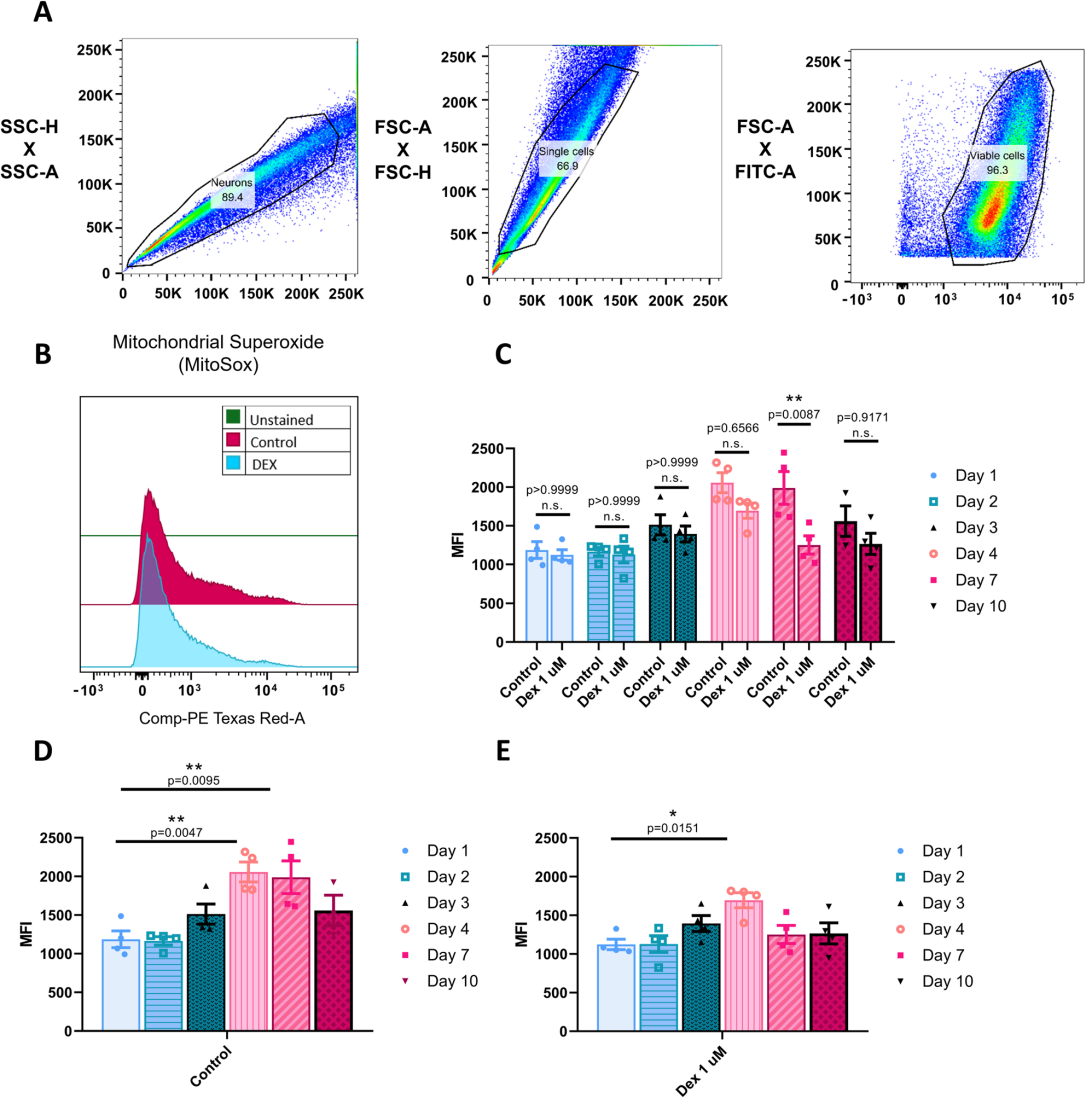
Dexmedetomidine reduced ROS production at day 7. (**A**) Gating strategy to select living neurons, stained with Calcein AM. (**B**) Representative MFI of mitochondrial superoxide production by neurons exposed to DEX compared to controls. (**C**) Quantification of MFI of mitochondrial ROS production by control and DEX-exposed cells at days 1, 2, 3, 4, 7 and 10. Control: D1 = 992 ± 108.7, D2 = 1007 ± 53.44, D3 = 1319 ± 129.4, D4 = 1828 ± 129.1, D7 = 1613 ± 212.0, D10 = 1245 ± 197.5 and DEX: D1 = 1034 ± 68.06, D2 = 823 ± 108.2, D3 = 1153 ± 103.3, D4 = 1401 ± 98.57, D7 = 1021 ± 117.2, D10 = 949 ± 136.6. Values are mean ± SEM F (5, 35) = 2.226, **p = 0.0087 by two-way ANOVA with Tukey’s post hoc analysis for multiple comparisons. (**D**) Quantification of MFI of mitochondrial ROS production on control cells at days 1, 2, 3, 4, 7 and 10. Control: D1 = 992 ± 108.7, D2 = 1007 ± 53.44, D3 = 1319 ± 129.4, D4 = 1828 ± 129.1, D7 = 1613 ± 212.0, D10 = 1245 ± 197.5 Values are mean ± SEM F (5, 17) = 7.313, P = 0.0008 by one-way ANOVA with Tukey’s post hoc analysis for multiple comparisons. (**E**) Quantification of MFI of mitochondrial ROS production on DEX-exposed cells at days 1, 2, 3, 4, 7 and 10. DEX: D1 = 1034 ± 68.06, D2 = 823 ± 108.2, D3 = 1153 ± 103.3, D4 = 1401 ± 98.57, D7 = 1021 ± 117.2, D10 = 949 ± 136.6. Values are mean ± SEM F (5, 18) = 3.968, P = 0.0133. n = 3–4 brains per condition.

ROS are important for various cellular functions, however an increase in their production is attributed to compromised cell health or death as an index of poor mitochondrial health. We thus sought to measure reactive oxygen species (ROS) production at Days 1, 2, 3, 4, 7 and 10 after DEX exposure and compare these data with the untreated control cultures. Using Flow-cytometry, we analyzed the populations of cells on different days that were double positive for Calcein AM (alive cells marker) (Fig. 5A) and MitoSox Red (cell producing ROS) and quantified the mean fluorescent intensity (MFI) (Fig. 5B) of the second marker in a manner analogous to that described previously by Brito de Monteiro et al.^37^. Control cells demonstrated a distinct ROS production profile over time whereas on days 1, 2 and 3, there was no significant variation in the MFI for MitoSox Red (Fig. 5D) (D1 mean = 992, SEM = 108.7, n = 4, D2 mean = 1007, SEM = 53.44, p > 0.9999, n = 4 and D3 mean = 1319, SEM = 129.4, p = 0.589, n = 4) indicating a healthy range of ROS. By day 4, there was a significant increase in the MFI (mean = 1828, SEM = 129.1, p = 0.0047, n = 4) which was maintained at day 7 (mean = 1613, SEM = 212.0, p = 0.0095, n = 4) however these values returned to normal by day 10 (mean = 1245, SEM = 197.5, p = 0.529, n = 3) suggesting a homeostatic pattern exhibited by healthy cells. Cells treated with DEX demonstrated a similar trend in ROS production (Fig. 5E) from days 1–4 (D1 mean = 1034, SEM = 68.06, n = 4, D2 mean = 823, SEM = 108.2, p > 0.9999, n = 4, D3 mean = 1153, SEM = 103.3, p = 0.498, n = 4 and D4 mean = 1401, SEM = 98.57, p = 0.015, n = 4). In contrast to the controls, the MFI for MitoSox Red, did not differ from D1 on D7, which was maintained at day 10 as well (D7 mean = 1021, SEM = 117.2, p = 0.0954, n = 4 and D10 mean = 949, SEM = 136.6, p = 0.93, n = 4). When we compared side by side controls versus DEX exposed cells (Fig. 5C), we saw a significant difference in the MFI at day 7 between controls and DEX (control: D7 mean = 1613, SEM = 212.0, n = 4 and DEX: D7 mean = 1021, SEM = 117.2, p = 0.0087, n = 4). These data can be correlated with the results shown in the previous assay where we saw an increase in the hyperfused mitochondrial fraction in the DEX-exposed cells suggesting that the DEX treated cells continued to exhibit a healthy ROS production regime in a manner analogous to that observed during early stages of cultures.

### DEX injected animals did not exhibit spatial or recognition learning and memory deficits

To test whether DEX exposure compromised learning and memory in animals, rat pups were injected with a concentration previously described in the literature^22^ and subsequently tested for learning and memory later in life. Specifically, we used P7 rat pups where 25 μg/kg DEX was administered subcutaneously either once (1×) or twice (2×) (second dose 24 h after the first dose). The animals were extensively monitored for their cardio-respiratory health, temperature, SpO_2_ and righting reflex. These parameters did not differ significantly from the control animals injected with saline (data not shown) as previously shown in the literature^22^. At P60, the animals underwent MWM and NORT testing.

**Figure 6:**
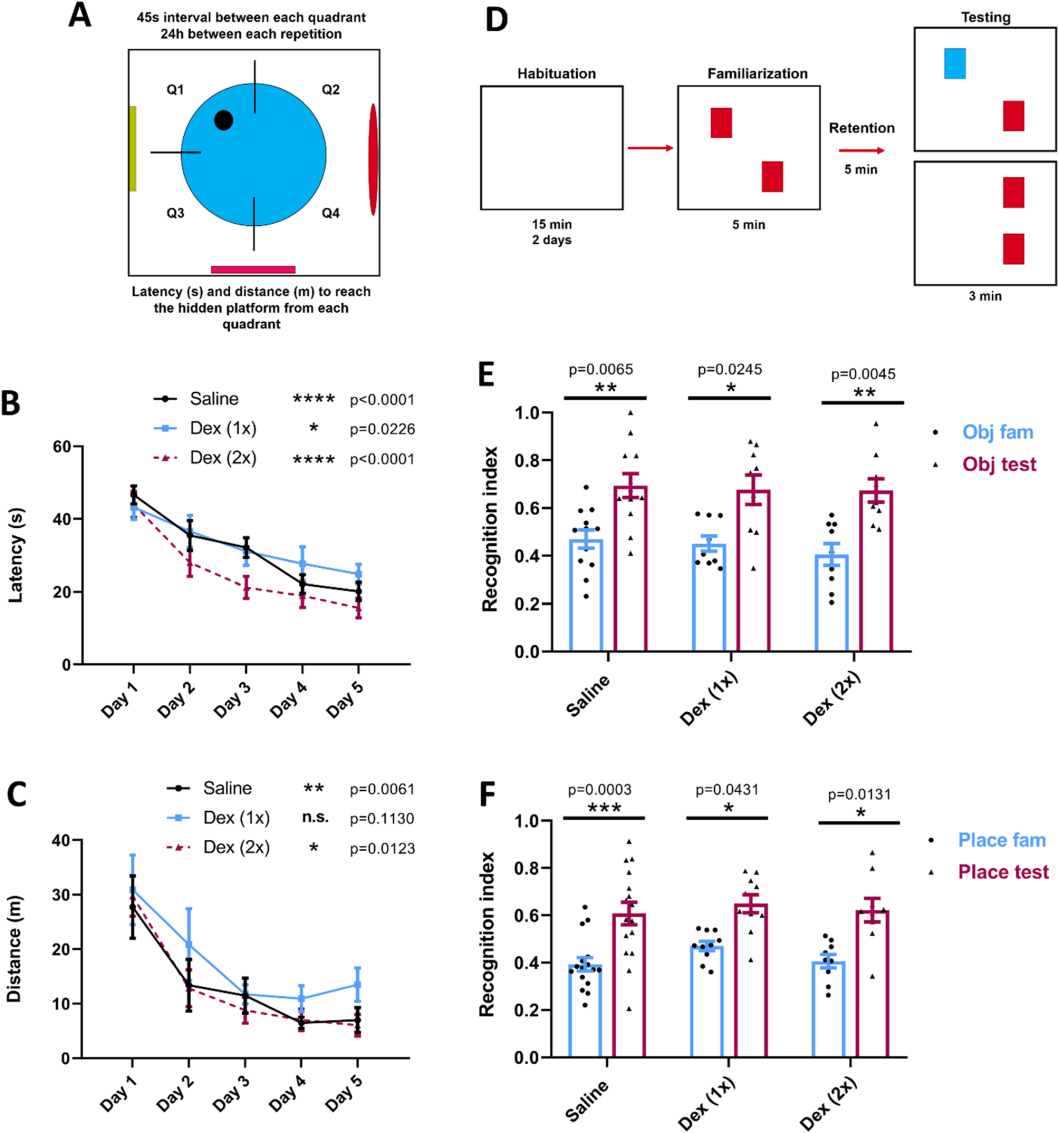
Dexmedetomidine did not affect cognitive capacities. (**A**) Schematic representation of Morris Water Maze protocol. (**B**) Quantification of the average latency between the 4 quadrants needed per day to reach the hidden platform; day 1: saline = 46.6 ± 2.448 (n = 16), Dex (1×) = 43.18 ± 3.325 (n = 11) and Dex (2×) = 43.99 ± 3.557 (n = 9) and day 5: saline = 20.14 ± 2.498 (n = 16), Dex (1×) = 24.83 ± 2.722 (n = 12) and Dex (2×) = 15.56 ± 2.695 (n = 9). Values are mean ± SEM F (8, 165) = 0.6369, *p = 0.0226, ****p < 0.0001 by one-way ANOVA with Tukey’s post hoc analysis for multiple comparisons. (**C**) Average swimming distance between the 4 quadrants required to reach the hidden platform; day 1: saline = 27.71 ± 5.711 (n = 13), Dex (1×) = 30.88 ± 6.356 (n = 11) and Dex (2×) = 29.74 ± 3.695 (n = 9) and day 5: saline = 6.994 ± 2.293 (n = 13), Dex (1×) = 13.48 ± 3.073 (n = 11) and Dex (2×) = 6.04 ± 1.987 (n = 9). Values are mean ± SEM F (8, 150) = 0.2167, *p = 0.0123, **p = 0.0061 by one-way ANOVA with Tukey’s post hoc analysis for multiple comparisons. (**D**) Schematic representation of Novel Object Recognition tests. (**E**) Recognition index calculated as the time spent with the novel object (TN) divided by the total time spent with both objects (TN + TF); familiarization: saline = 0.4698 ± 0.0384 (n = 11), Dex (1×) = 0.451 ± 0.0322 (n = 9) and Dex (2×) = 0.405 ± 0.0457 (n = 9) and testing: saline = 0.694 ± 0.0496 (n = 12), Dex (1×) = 0.677 ± 0.0624 (n = 9) and Dex (2×) = 0.674 ± 0.04815 (n = 9). Values are mean ± SEM F (2, 54) = 0.136, *p = 0.0245, **p = 0.045 and **p = 0.0065 by one-way ANOVA with Tukey’s post hoc analysis for multiple comparisons. (**F**) Recognition index calculated as the time spent with the object located in a different place (TN) divided by the total time spent with both objects (TN + TF); familiarization: saline = 0.393 ± 0.0283 (n = 16), Dex (1×) = 0.471 ± 0.01996 (n = 10) and Dex (2×) = 0.406 ± 0.0285 (n = 9) and testing: saline = 0.608 ± 0.0475 (n = 16), Dex (1×) = 0.649 ± 0.038 (n = 10) and Dex (2×) = 0.622 ± 0.0502 (n = 9). Values are mean ± SEM F (2, 64) = 0.1315, *p = 0.0131 and *p = 0.043, ***p = 0.0003 by one-way ANOVA with Tukey’s post hoc analysis for multiple comparisons. Bars indicate ± SEM.

For MWM, the animals were placed in a pool to reach a hidden platform invoking different spatial cues that were placed on the walls of the room for a period of 5 consecutive days (Fig. 6A). The average latency (time required to reach the platform once they were placed in the water) (Fig. 6B) and the distance traveled (Fig. 6C) before reaching the hidden platform were measured for each trial. One-way ANOVA statistical analysis was performed to compare the average latency and distance from day 1 to day 5. In terms of latency, we saw a statistically significant improvement in the time required to reach the platform comparing days 1 and 5 for both DEX-treated animals that were injected either once (1×) (Day 1: mean = 43.18, SEM = 3.325 n = 11 and Day 5: mean = 24.83, SEM = 2.722, p = 0.023 n = 11) or twice (2×) (Day 1: mean = 43.99, SEM = 3.557, n = 9 and Day 5: mean = 15.56, SEM = 2.695, p < 0.0001, n = 9) as well as controls (Day 1: mean = 46.6, SEM = 2.448, n = 16 and Day 5: mean = 20.14, SEM = 2.498, p = 0.006, n = 16) (Fig. 6B), suggesting that spatial learning had occurred in all groups. Regarding the distance swum by the animals, there was not a significant improvement between days 1 and 5 in the 1×-injected animals (Day 1: mean = 30.88, SEM = 6.356, n = 11 and Day 5: mean = 13.48, SEM = 3.073, p = 0.113, n = 11), which meant that this cohort of animals, in particular, swam longer distances in the pool to locate the platform as compared with saline and 2× DEX injected animals. Nevertheless, these animals still located the platform within a time window similar to that of the controls.

For the NORT experimental paradigm (Fig. 6D), we measured the Recognition index [RI = TN/(TN + TF)] where TN = time spent while exploring the new object or the object placed at a different location and TF = time spent exploring the familiar object. In all groups, both for the introduction of the novel object (Fig. 6E) and the novel location of the familiar object (Fig. 6F), there was a significant increase in the time spent interacting with the novel object (introduction of novel object: control: mean = 0.694, SEM = 0.0496, p = 0.0065, n = 12, DEX 1×: mean = 0.677, SEM = 0.0624, p = 0.0245, n = 9 and DEX 2×: mean = 0.674, SEM = 0.04815, p = 0.0045, n = 9 and Novel location: control: mean = 0.608, SEM = 0.0475, p = 0.0003, n = 16, DEX 1×: mean = 0.649, SEM = 0.038, p = 0.043, n = 10 and DEX 2×: mean = 0.622, SEM = 0.0502, p = 0.0131, n = 9) as compared to the familiarization phase where the ratio was closer to 0.5 (Introduction of novel object: control: mean = 0.4698, SEM = 0.0384, n = 12, DEX 1×: mean = 0.451, SEM = 0.032, p = 0.0245, n = 9 and DEX 2×: mean = 0.405, SEM = 0.046, p = 0.0045, n = 9 and Novel location: control: mean = 0.393, SEM = 0.0283, n = 16, DEX 1×: mean = 0.471, SEM = 0.01996, n = 10 and DEX 2×: mean = 0.406, SEM = 0.0285, n = 9).

Taken together, these data show that DEX did not compromise learning and memory when administered to young pups, thus further underscoring its safety for clinical use.

## Discussion

Most previous studies have shown DEX not only to be non-toxic but also neuroprotective against cytotoxicity induced by other anesthetics^17,18,20^. However, there exist controversies vis-à-vis its neuroprotective nature in situ^17,19^ and also at the behavioral levels^18,19,42^. Moreover, the mechanisms also remain poorly defined, specifically in the context of its effects on neuronal growth and synaptic structure assembly at the level of the individual neuron—due mainly to the complex nature of the intact brain or intact brain slices used in other studies.

Although DEX can be used in combination with magnesium sulfate (MgSO_4_) in the clinical setting^43,44^, we have only assessed DEX individually, as MgSO_4_ has been reported to have neuroprotective properties^45^ whereas we aimed to test DEX’s properties on neuronal health on its own. This study provides the first direct and unequivocal evidence for the non-neurotoxic nature of DEX when tested for cellular viability, neurite outgrowth and synaptic network assembly. Moreover, we have demonstrated for the first time, that unlike other conventionally used anesthetic agents, which exert their neurotoxic effects by perturbing mitochondrial structure and function, DEX instead promotes mitochondrial health while maintaining a healthy ROS production. These results both endorse our previous finding that anesthetic-induced toxicity may involve the mitochondria while strongly suggesting that DEX-mediated positive effects on neuronal health may also involve this organelle.

Notwithstanding their essential requirements for most surgical procedures, the neurotoxic nature of various anesthetic agents, as deduced from numerous animal studies, cannot be overlooked. However, the investigation of the underlying neurotoxic mechanisms cannot be conducted in any human studies at the level of individual neurons—owing primarily to the experimental logistics and the ethical implications. While some studies do not support detrimental effects of anesthetics in humans^46,47^, a few others have suggested possible anesthetic-induced toxicity in both developing^48,49^and aging brains^50,51^. These studies, together with research on non-human primates^52–56^, have raised significant red flags thus compelling healthcare regulatory agencies to recommend that warning labels be placed on anesthetics when given to pregnant mothers and young children^57^. Moreover, in recent years, several studies have urged that we take into consideration the potential neurotoxic effects of anesthetic agents on brain development in humans and define the underlying mechanisms using animal models^5,57,58^. Despite numerous attempts to understand the effects of anesthetics on neuronal homeostasis, the results remain either equivocal or controversial^5^.

In contrast to common conventional anesthetics, DEX is a newer agent being used both as a sedative and analgesic^59^ agent that does not rely on NMDA or GABAA receptor function^60^. DEX acts on the alpha2 adrenoreceptor, acting as an agonist^59^, and some of its physiological functions include the induction of sedation, pain modification, inhibition of insulin release and the presynaptic inhibition of norepinephrine release^60^.

Although DEX is currently used in pediatric medicine^61,62^, there are also studies demonstrating that higher clinical concentrations of DEX indeed cause cell death in situ^25,63^. It would therefore appear that under certain experimental conditions DEX may induce neurotoxicity in brain slices and these subsequent effects may involve a variety of signalling pathways. However, in many other instances, DEX appears not only to be non-neurotoxic but also neuroprotective against the toxicity induced by other anesthetic agents such as sevoflurane, propofol or ketamine^22,64,65^. Previous studies have shown a number of anesthetic agents affect synaptic structures and function^33,66,67^; however, similar conclusive evidence for DEX is missing. This study is thus the first to demonstrate that although DEX enhanced neurite outgrowth, it did not affect the total number of synaptic connections formed between the cortical neurons. When examined in light of previous studies where DEX-induced positive effects were shown to involve the PI3K/Akt/GSK3β pathway^18,20,42^ invoking growth promoting factors (insulin for example), it therefore stands to reason that this anesthetic may activate signaling that could potentially be growth permissive. However, in our study, we found that notwithstanding this enhanced growth, neurons did not make additional synaptic contacts as compared with their control cohort.

Even though several studies have vouched for DEX safety, the evidence is not unequivocal. For instance, on the one hand, Liu et al.^25^ tested different concentrations of DEX ranging from 0.001 to 0.2 µM, and found significant cell death in a dose-dependent manner. On the other hand, Sanders et al.^24^ demonstrated that when cultured neurons that were previously treated with wortmannin and staurosporine (to promote neuroapoptosis) were exposed to 0.1–100 µM DEX, it rescued neurons from cell death in a dose-dependent manner^24^. In the present study however, we generated a dose response curve for DEX, ranging from 0.01 to 10 µM and identified a concentration of 1 µM to be the least cytotoxic. As this concentration is generally used in the clinic^39^, our data thus endorse it to be potentially safer in humans. Data presented in this study are also consistent with earlier work completed by Wu et al. which demonstrated that DEX protected neurons against apoptosis, axonal degradation, and synaptic degeneration after traumatic brain injury in a murine model^28^. Although Wu et al.^63^ did not show direct effects of DEX on cellular viability, they did nevertheless demonstrate the extent to which it rescued neurons from axonal degradation and synaptic degeneration in the intact animal. The rationale for using DEX in a traumatic brain injury model used by Wu et al.^28^, where the induced damage is often inconsistent and unreliable remains unclear, however they did nevertheless find this anesthetic to be neuroprotective. The present study provides direct evidence that in a simple model system, which is devoid of whole animal complexity and inconsistent experimental paradigm (i.e. methods to induce traumatic injury), DEX prevented neurons from cell death and promoted neurite outgrowth (increased the neurite number and total length). These data thus confirm results obtained from in vivo studies and provide further unequivocal support at the resolution of individual neurons and synaptic structures. We suggest that these growth permissive or inductive aspects of DEX are likely attributable to its positive effects on mitochondrial structures, function and production of a healthy ROS regime.

Notwithstanding the fact that DEX exerted growth permissive effects on neurites of cultured neurons, it did not however affect the total number of synaptic puncta between the two connecting neurons during the time window the cells were fixed (Day 7). It needs to be noted that most synapses that form between these neurons are thus consistent and remain unchanged even when cells are examined after 3 weeks. This argument is supported by several previous studies which showed that if in vitro neurons remained unperturbed due either the induction of electrical activity or pharmacological perturbations that the number of synapses formed between neurons remained unchanged for up to 100 days^68–70^. Whereas, at the cellular level, it would be almost impossible to demonstrate unequivocally that either the total number of persisting synapses, or the efficacy of an individual synapse would have remained consistent at any given time, an extensive study conducted at the proteomics level showed that the turnover of new synaptic proteins occurred primarily after the neuronal stimulation^71^. This rules out the possibility that DEX might tip the balance towards exuberant synaptic connectivity thus perturbing the innate balance of the synaptic “real-estate” that is shared between any two interconnected neurons. In contrast to these findings, previous studies have shown that both local and general anesthetics reduce the total number of synaptic puncta, as shown by a decrease in the relative expression of synaptophysin after isoflurane exposure^72^. It is therefore feasible that the neuroprotective effects of DEX observed in the Wu et al.^28^ study may not have much to do with its role as an anesthetic, but rather it may have acted in a manner analogous to that of ketamine^34,73,74^. Specifically, their growth permissive or neuroprotective effects may have involved signaling mechanisms other then their modes of actions as an anesthetic agent^72^.

An important finding of this study is the link between DEX and its role in modifying mitochondrial networks. Specifically, we found that DEX exposure increased the fused mitochondrial fraction population in the neurons as compared with the intermediate fraction, without significantly modifying their fragmented form. Any prolonged imbalance in the fission/fusion equilibrium is widely documented to be detrimental to cellular health and viability, and has been shown to compromise key functions such as growth cone dynamics^75^, axonal regeneration, branching, and synapse formation^75–78^.

Moreover, this study provided for the first time a comprehensive analysis regarding ROS production over time in cultured neurons. We found that DEX decreased ROS production over several days which could be the potential driver underlying its impact on neuronal health; this would however need to be tested experimentally in the future. Our data is nevertheless consistent with previous studies where DEX was shown to diminish ROS production in non-neuronal tissue such as kidney^79^ and cardiomyocytes^80^, as well as following cerebral ischemia^81^.

As neurons used in our cultures were primarily from rat cortices, which we found to respond to anesthetics in a manner analogous to hippocampal neurons (data not shown), it could be argued that adrenergic receptors may have been absent and that this could account for the lack of evidence of DEX toxicity. To rule out this possibility, we injected rat pups for in vivo experiments, thus exposing the entire brain to DEX. These animals did not demonstrate any deficits in learning and memory, providing further support that DEX is non-neurotoxic when used in clinically relevant concentrations. Duan et al.^19^ have previously demonstrated that when P7 pups (female only) were injected with DEX at a concentration similar to the one used in this study, their spatial memory tested in the Morris Water Maze^19^ remained indistinguishable from the controls. In the present study, not only did we use one, but also two injections in a space of 24 h involving both male and female rats and invoked both Morris Water Maze and NORT learning and memory paradigms. The data presented here thus further endorses our in vitro results underscoring the non-neurotoxic nature of DEX and supports its utility in the clinical realm.
